# Obesity and Oral Health: The Link Between Adipokines and Periodontitis

**DOI:** 10.17925/EE.2024.20.1.7

**Published:** 2024-01-25

**Authors:** Ana Checa-Ros, Wei-Chung Hsueh, Belén Merck, Henry González-Torres, Valmore Bermúdez, Luis D’Marco

**Affiliations:** 1. Grupo de Investigación en Enfermedades Cardiorrenales y Metabólicas, Departamento de Medicina y Cirugía, Facultad de Ciencias de la Salud, Universidad Cardenal Herrera-CEU, CEU Universities, Valencia, Spain; 2. Departamento de Odontología, Facultad de Ciencias de la Salud, Universidad Cardenal Herrera-CEU, CEU Universities, Valencia, Spain; 3. Facultad de Ciencias de la Salud, Universidad Simón Bolívar, Barranquilla, Colombia; 4. Centro de Investigaciones en Ciencias de la Vida, Universidad Simón Bolívar, Barranquilla, Colombia

**Keywords:** Adipokines, inflammation, obesity, oral health, periodontitis

## Abstract

Periodontitis is a chronic inflammatory disease of the periodontium, or the supportive tissues around the tooth. This disease has been related to different risk factors, such as the presence of plaque and calculus, tobacco smoking, low socioeconomic status, and the immune state of the host. Importantly, the chronic inflammatory environment generated by periodontitis may lead to tooth loss and diverse systemic complications, such as cardiovascular disease, osteoarthritis and metabolic disease. Recent investigations have supported the role of obesity as a risk factor for periodontitis. Furthermore, studies have found obesity to compromise healing after periodontal therapy; however, the mechanisms underlying this association are not well understood. Proteins called 'adipokines' could be the factor linking obesity to periodontitis. Adipokines are bioactive molecules with hormonal properties and a structure similar to cytokines produced by the adipose tissue. Although adipokines have both pro-and anti-inflammatory effects, the shift towards pro-inflammatory actions occurs when the adipose tissue becomes pathological, as observe in the progression of conditions such as obesity or adiposopathy. This article reviews the role of adipokines in the pathophysiology and progression of periodontitis by focusing on their impact on inflammation and the molecular mechanisms through which adipokines contribute to the onset and development of periodontitis.

Periodontitis is a chronic inflammatory disease of the periodontium, or the supportive tissues around the tooth, which includes the gingival tissue, alveolar bone, cementum and the periodontal ligament.^1^ Caused by pathogenic bacteria, this disease has been recognized for at least 5,000 years and has been related to diverse risk factors, such as the presence of plaque and calculus, tobacco smoking, low socioeconomic status, and the immune state of the host. Periodontitis can be successfully treated with non-surgical options (scaling and root planning, local and systemic chemotherapy), as well as surgical therapies in refractory cases.^1^ However, the chronic inflammatory environment generated by this disease may lead to tooth loss in around 14% of patients and to diverse systemic complications, such as cardiovascular disease (CVD), osteoarthritis and type 2 diabetes.^2,3^

From a physiopathological point of view, the bacterial presence induces a host immune-inflammatory response that can lead to irreversible bone-matrix degradation and resorption. Thus, the chronic nature of periodontitis is associated with a multifactorial immuno-inflammatory process involving the periodontium.^4^ In view of this, a common inflammatory background between periodontitis and other chronic diseases, such as CVD, diabetes, rheumatoid arthritis and Alzheimer's disease, has been suggested.^5^

Recent investigations support the role of obesity as a risk factor for periodontitis.^6^ Likewise, studies have found obesity to compromise healing after periodontal therapy.^7^ Obesity is one of the components of metabolic syndrome, a systemic multifactorial condition including type 2 diabetes, hypertension and dyslipidaemia.^8^ The mechanisms underlying the association between obesity and the conditions causing metabolic syndrome are not well understood. However, it seems that the adipose tissue surrounding visceral organs (visceral adiposity) and this tissue's production of specific proteins, called 'adipokines', could represent the metabolic-syndrome-related link to periodontitis.^9^ Adipokines are bioactive molecules with hormonal properties and a structure similar to cytokines that are produced by the adipose tissue. They provide the host with information on long-term energy storage by affecting the sensitivity of peripheral tissues to insulin.^10^ Moreover, they significantly influence several physiological processes, including immunological response, blood pressure control, reproductive function and energy balance.^10,11^ Adipokines also have effects on the inflammatory and healing processes, playing an essential role in fusing systemic metabolism with the immune function.^12^ Although different adipokines have both pro-and anti-inflammatory effects, the balance shifts towards pro-inflammatory actions when the adipose tissue turns pathological, as seen in obesity or the other systemic conditions associated with metabolic syndrome.^13^ This article reviews the role of adipokines in the pathophysiology and progression of periodontitis.

## Inflammatory mechanisms behind periodontitis

Periodontal disease starts with the formation of a dental biofilm in an ordered (layer-to-l ayer) sequence, which represents the bacterial colonization enclosed by a protective matrix composed of polysaccharides and glycoproteins.^14^ The initial bacteria contributing to the biofilm are facultative gram-positive bacteria, such as the *Actinomyces* and *Streptococcus* species.^15^ The progressive deposition of layers gradually leads to an oxygen-deficient environment that favours the appearance of anaerobic bacteria, such as the *Fusobacterium* and *Prevotella* species, causing dysbiosis of the periodontal microbiota.^16^

The bacterial secretion of lipopolysaccharides (LPS) activates the innate immune response, with the production of proinflammatory cytokines by macrophages, such as interleukin-1 beta (IL-1β), tumour necrosis factor-alpha (TNF-α) and interleukin-6 (IL-6).^16^ In a further step, the adaptive immune response is triggered, activating B and T cells and leading to the inflammatory cascade.^17^ This phenomenon clinically translates into erythematous, bleeding and inflamed gingiva.

Depending on the host's immunological response and dental factors, such as the position of the tooth and the crown–root ratio, the process can progress to bone loss by stimulating bone resorption and influencing bone regeneration.^18^ Osteoclastogenesis is activated directly by stimulating osteoclasts through the activation of receptor activator of nuclear factor-κB ligand (RANKL) by T and B cells and indirectly with the release of chemokines that disrupt the action of osteoblasts or osteoblast precursors, such as C-X-C motif chemokine ligand 10 (CXCL10), C-X-C motif chemokine ligand 12 (CXCL12), C-X-C motif chemokine ligand 13 (CXCL13) and C-C motif chemokine ligand 5 (CCL5).^17,19^

## Adipokines and inflammation

**Figure 1:**
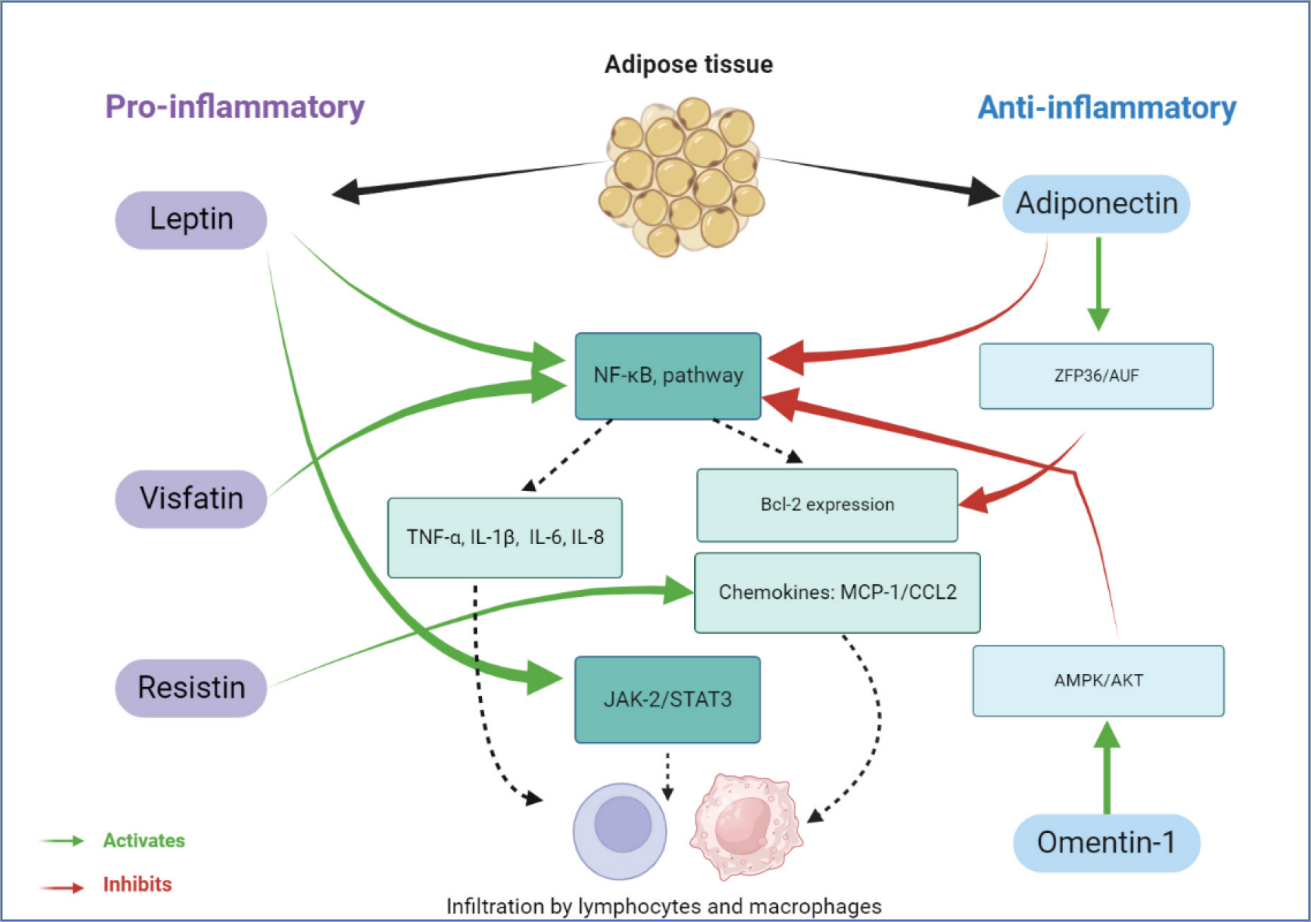
Role of the adipose tissue in the inflammation process via the secretion of adipokines with proinflammatory (in purple) and anti-inflammatory actions (in blue)

Adipokines are low-molecular-weight and pharmacologically active proteins that possess pleiotropic activity. Acting in the hypothalamic region as orexigenic and anorexigenic hormones, adipokines play a crucial role in energy metabolism by communicating the nutrient status of the organism.^20^ Although the role of adipokines was initially thought to be restricted to metabolic activities (the regulation of glucose and lipid metabolism), adipokines are currently considered key players in the complex network of soluble mediators involved in the pathophysiology of immune-inflammatory diseases. Recent scientific investigations have focused on the potential influence of visceral adipose tissue as a source of adipokine secretion and inflammation (*Figure 1*).

Leptin was the first member of the adipokine family to be identified. Its levels depend on the size and number of adipocytes. Leptin centrally regulates body weight by linking nutritional status and neuroendocrine function.^21^ It is considered a proinflammatory adipokine: the binding to its receptor upregulates a number of signalling pathways, such as Janus kinase-2 (JAK-2)/signal transducer and activator of transcription-3 (STAT3).^21^ Leptin also stimulates the secretion of proinflammatory cytokines, such as TNF-α and IL-6; this process is associated with insulin resistance and type 2 diabetes.^22^

*AKT = protein kinase B; AMPK = 5' adenosine monophosphate-activated protein kinase; AUF-1 = ARE/poly(U)-binding/degradation factor-1; Bcl-2 = B-cell lymphoma 2; CCL2 = chemokine ligand 2; IL-1β = interleukin-1 beta; IL-6 = interleukin-6; IL-8 = interleukin-8; JAK-2 = Janus kinase-2; MCP-1 = monocyte chemoattractant protein-1; NF-κB = nuclear factor kappa-light-chain-enhancer of activated B cells; STAT3 = signal transducer and activator of transcription-3; TNF-α = tumour necrosis factor-alpha; ZFP36 = zinc finger protein 36.*

**Figure 2:**
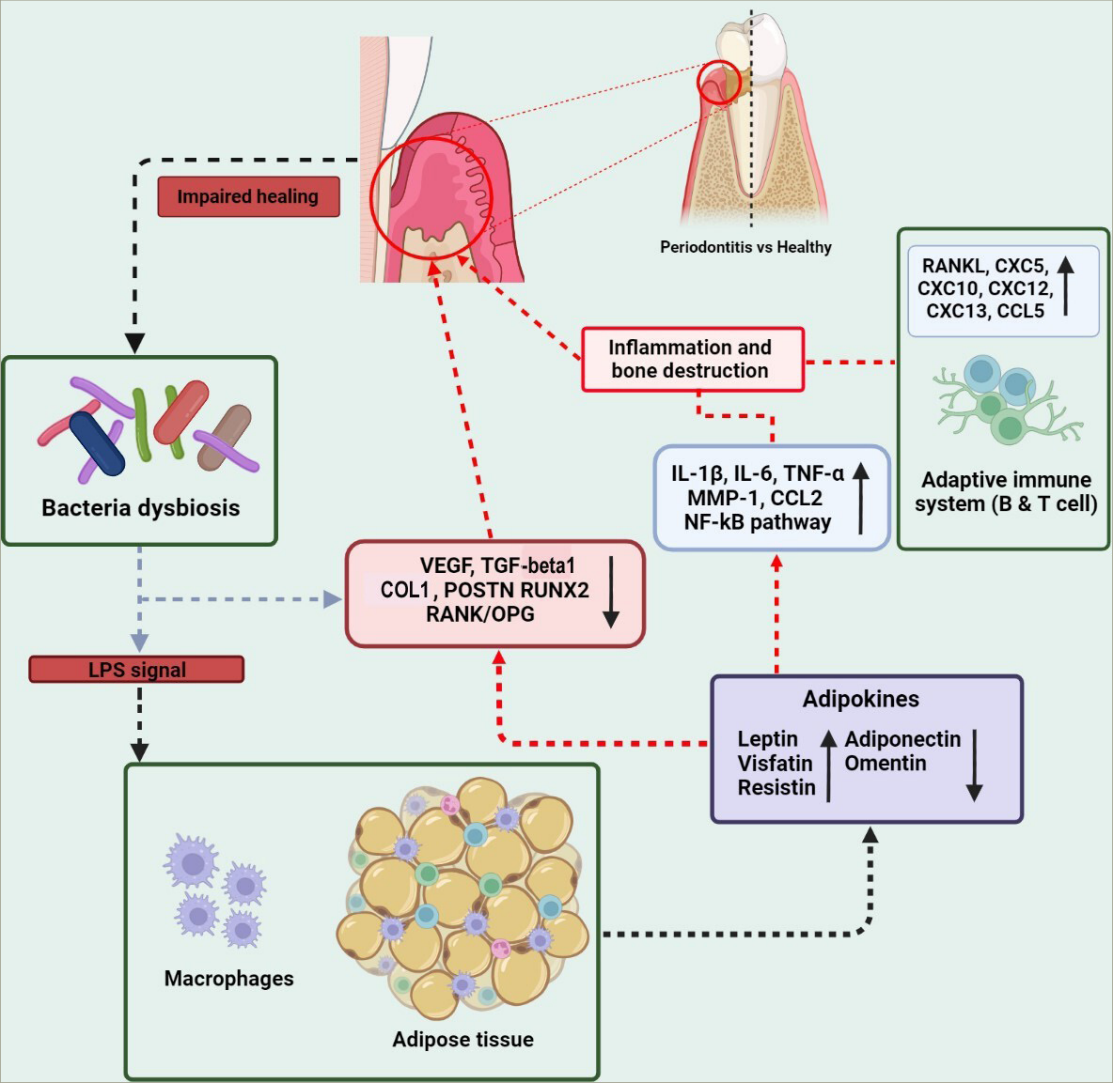
Inflammatory molecular mechanisms of adipokines contributing to periodontitis

*CCL2 = chemokine ligand 2; CCL5 = C-C motif ligand 5; COL1 = collagen type 1; CXC = C-X-C motif chemokine ligand; IL-1β = interleukin-1 beta; IL-6 = interleukin-6; LPS = lipopolysaccharide; MMP-1 = matrix metalloproteinase-1; NF-κB = nuclear factor kappa-light-chain-enhancer of activated B cells; POSTN = periostin; OPG = osteoprotegerin; RANK = receptor activator of NF-κB; RANKL = RANK ligand; RUNX2 = runt-related transcription factor-2; TGF-beta1 = transforming growth factor-beta 1; TNF-α = tumour necrosis factor-alpha; VEGF = vascular endothelial growth factor; vs = versus.*

Adiponectin is a 244-amino acid protein produced by adipocytes that binds to specific receptors, AdipoR1 and AdipoR2.^23^ It abolishes the LPS-induced nuclear factor kappa-l ight-chain-enhancer of activated B cells (NF-κB) translocation, suppressing the LPS-stimulated expression of IL-1β, IL-6, and interleukin-8 (IL-8).^24^ Adiponectin also activates the expression of zinc finger protein 36 (ZFP36) and ARE/poly(U)-binding/ degradation factor-1 (AUF1), which favours beclin-1-activated autophagy in macrophages by destabilizing the complex formed by B-cell lymphoma 2 (Bcl-2) and Beclin-1.^25^

Visfatin, a protein hormone with insulin-l ike effects mainly produced by adipocytes and macrophages, plays a key role during inflammatory processes.^26^ Plasma levels of visfatin are raised in several diseases: for example, type 2 diabetes, obesity, metabolic syndrome, atherosclerosis, cancer, rheumatoid arthritis and sepsis.^27–31^ Visfatin exhibits inflammatory properties by inducing the production of IL-1β, TNF-α, and IL-6 by monocytes and upregulating IL-6 gene expression in human endothelial cells.^26,32^

**Table 1:** Adipokines and periodontal disease

Adipokine	Role	Mechanism of action	Highlights
OM-1	Anti-inflammatory	1. Salivary OM-1 is a remarkable inhibitor of inflammation, acting through multiple cellular signalling pathways and molecular mechanisms. 2. It effectively blocks TNF-α induced by cyclooxygenase-2, superoxide production and the expression of adhesion molecules in endothelial cells, thus disrupting the ERK/NF-κB pathway. 3. It exerts its anti-inflammatory effects by directly suppressing the expression of proinflammatory mediators, including TNF-α, IL-6 and monocyte chemotactic protein-1, through the AMPK/ AKT pathway. 4. OM-1 promotes signalling pathways that stimulate the proliferation of human osteoblasts, suggesting that it plays a significant role as a regulator of bone remodelling	1. Lower salivary OM-1 levels were identified in patients with chronic periodontitis and associated with increased levels of periodontal parameters. 2. Higher salivary OM-1 levels after non-surgical periodontal therapy were also concomitant with an improvement in the periodontal health status. 3. It may be concluded that OM-1 has an anti-inflammatory role in periodontitis, and its expression may have a potential role in the immunopathogenesis of chronic periodontitis
APN	Anti-inflammatory	1. APN, when binding to its receptors, exhibits strong anti-inflammatory effects. It can counteract proinflammatory responses in different cell types. For instance, APN increases the expression of chemokines in epithelial cells through NF-κB signalling but paradoxically prevents LPS-induced NF-κB nuclear translocation, thereby inhibiting the expression of inflammatory cytokines such as IL-1β, IL-6 and IL-8 in human GECs. 2. APN effects are also influenced by the context; it initially promotes TNF-α production through the ERK1/2→EGR-1 and NF-κB-dependent pathways, subsequently boosting IL-10 expression, which dampens the inflammatory response in LPS-exposed macrophages. 3. This adipokine contributes to bone metabolism by enhancing osteoblast recruitment, differentiation and proliferation while inhibiting osteoclast differentiation and activity	1. APN alleviates periodontitis partly due to its action in inflammation and bone and can mediate different stages of bone metabolism. 2. The level of APN has an inverse association with periodontitis. 3. The underlying mechanisms may include the functions of APN in suppressing inflammation and promoting bone regeneration
Resistin	Proinflammatory	1. Resistin is expressed mainly by macrophages in response to bacterial and inflammatory challenges, suggesting that it plays a role in inflammation. Moreover, it plays a role in bone remodelling as it enhances the proliferation of preosteoblast cells and in osteoclastogenesis since it increases the number of differentiated osteoclasts. 2. Resistin expression in PDL cells is upregulated in the presence of the proinflammatory mediator IL-1β. Thus, resistin has the potential to increase the expression of IL-6 and IL-8 in periodontal cells. 3. In addition, resistin induces downregulation of hard tissue (BMP2, RUNX2 and OCN) and matrix (POSTN and COL1) markers and growth factors (TGF-β1 and VEGF) in periodontal cells	1. Resistin is produced by periodontal cells and tissues. 2. Microbial and inflammatory stimuli increase resistin expression and production in the periodontium. 3. Resistin seems to interfere with soft and hard tissue metabolism by reducing alkaline phosphatase activity and markers related to bone tissue and matrix formation
Leptin	Proinflammatory	1. The decrease in leptin is associated with an increase in VEGF. This inverse correlation between leptin and VEGF in human gingiva supports that leptin has no pro-angiogenic effects on periodontal tissues. 2. TGF-β1, another important growth factor, comprises three isoforms and promotes wound healing by its stimulatory effects on the migration, chemotaxis, and proliferation of monocytes/macrophages, fibroblasts, and endothelial cells and keratinocyte migration and re-epithelialization. 3. Leptin abrogated the stimulatory actions of EMD on the aforementioned growth and transcription factors as well as matrix molecules, suggesting that leptin may interfere with the EMD-induced effects on both periodontal soft and hard tissue regeneration	1. Leptin production is under the control of the obesity gene. It is an adipokine entirely produced in the adipocytes, with the fundamental function of controlling appetite and regulating food intake and energy expenditure. 2. Leptin negatively interferes with the regenerative capacity of PDL cells, suggesting leptin as a pathomechanistic link between obesity and compromised periodontal healing
Visfatin	Proinflammatory	1. Visfatin is thought to have insulin-l ike effects, particularly those that lower plasma glucose levels. 2. The relationship between the serum and GCF concentrations of visfatin and periodontal diseases has been evaluated, concluding that visfatin concentration increased with disease severity in the serum and GCF. 3. Higher expression of visfatin, NF-κB, PI3k, TNF-α, and IL-1β in patients with gingivitis and periodontitis suggest that increased visfatin levels play a role in the pathogenesis of periodontitis. 4. TNF-α and IL-6, which are known as tissue destruction mediators, function as proinflammatory cytokines, and their levels increase during inflammation. 5. In patients with severe periodontitis, corpuscles and adipocytes in the liver have been proposed as sources of IL-6. This information helps us to understand the high level of GCF IL-6 in individuals who are obese	1. Higher expression of visfatin is observed in patients with gingivitis and periodontitis. 2. Visfatin and IL-6 levels might play a role in the pathogenesis of periodontal disease and can be used as reliable markers for monitoring the course of periodontitis. 3. Visfatin may accelerate periodontal inflammation and bone destruction via the production of MMP-1 and CCL2

*AKT = protein kinase B; AMPK = 5' adenosine monophosphate-activated protein kinase; APN = adiponectin; Bcl-2 = B-cell lymphoma 2; CCL2 = chemokine ligand 2; COL1 = collagen type 1; EGR-1 = early growth response-1; EMD = enamel matrix derivative; ERK = extracellular signal-regulated protein kinase; GCF = gingival crevicular fluid; GEC = gingival epithelial cell; IL = interleukin; LPS = lipopolysaccharide; MMP-1 = matrix metalloproteinase-1; NF-κB = nuclear factor kappa-light-chain-enhancer of activated B cells; OCN = osteocalcin; OM-1 = omentin-1; OPG = osteoprotegerin; PDL = periodontal ligament; PI3k = phosphatidylinositol 3-kinase; POSTN = periostin; RANK = receptor activator of NF-κB; RUNX2 = runt-related transcription factor-2; TGF-β1 = transforming growth factor-beta 1; TNF-α = tumour necrosis factor-alpha; VEGF = vascular endothelial growth factor; ZFP36 = zinc finger protein 36.*

Resistin is a cysteine-rich secretory protein prominently produced by macrophages and monocytes in response to inflammatory challenges. The secretion of resistin is influenced by certain metabolic and inflammatory diseases, such as CVD, atherosclerosis, type 2 diabetes and insulin resistance.^33,34^ Resistin is known to stimulate angiogenesis and the synthesis of proinflammatory cytokines and chemokines via the induction of C-C motif ligand 2 (CCL2) and monocyte chemoattractant protein-1 (MCP-1).^35,36^

Omentin-1 is a novel anti-inflammatory adipokine produced in the adipose tissue, inhibiting the production of superoxide and adhesion molecules in endothelial cells and blocks the extracellular signal-regulated protein kinase (ERK)/NF-κB pathway.^37,38^ In addition, omentin-1 can exert inflammatory modulation by promoting the AMP-activated protein kinase (AMPK)/protein kinase B (AKT) pathway, leading to the downregulation of proinflammatory mediators, such as TNF-α, IL-6 and MCP-1.^39^ Moreover, omentin-1 has been involved in bone remodelling by stimulating the phosphatidylinositol 3-kinase (PI3K)/AKT signalling pathway, which promotes the proliferation of human osteoblasts.^40^

All these adipokines are dysregulated in obesity, seemingly contributing to a low-grade inflammatory state through endocrine, paracrine, autocrine and juxtacrine crosstalk mechanisms.^41^ Altogether, adipokines have emerged as biomarkers and therapeutic targets for immune disorders. However, given the complexity of the role played by the adipokine network in the pathogenesis and progression of inflammatory diseases, it is still unclear whether it is possible to target the mechanism(s) by which adipokines contribute to disease without suppressing their physiological functions. Further insights into how adipokines affect the immune system and contribute to the development of inflammatory-associated disorders and diseases are needed for developing novel therapeutic approaches.

## Adipokines and periodontitis

Researchers have suggested that adiponectin plays a potential role in tissue regeneration by producing anti-inflammatory factors to promote cell proliferation and differentiation. In the bone, for instance, adiponectin promotes the recruitment and differentiation of osteoblast progenitors, inhibiting the activity of osteoclasts.^42^ In periodontal ligament cells, adiponectin inhibits the expression of TNF-α and *Actinobacillus actinomycetemcomitans*, which is highly associated with periodontitis.^43^ Furthermore, adiponectin counteracts the stimulatory effects of *Porphyromonas gingivalis* by decreasing the expression of proinflammatory cytokines, such as IL-1β, IL-6, IL-8, and the matrix metalloproteinases (MMPs) MMP-1 and MMP-3.^24,42^ At the same time, adiponectin induces the production of anti-inflammatory molecules, such as interleukin-10 and heme oxygenase-1 (HO-1). Studies have indicated that adiponectin plays a potential prevention role in periodontal infection by blocking the nuclear translocation of NF-kB and decreasing the expression of IL-6 and IL-8.^44^ This adipokine also promotes periodontal healing by upregulating the expression of transforming growth factor-beta 1 (TGF-β1), vascular endothelial growth factor (VEGF) and periostin (POSTN).^45^

Interestingly, periodontitis can increase plasma leptin levels, which decrease after periodontal therapy, highlighting the primary proinflammatory properties of this protein.^46,47^ Leptin secretion is stimulated by *Prevotella intermedia* LPS, resulting in the upregulation of TNF-α.^48^ In addition, researchers have suggested that leptin may have a potential impact on periodontal proper healing and regeneration by downregulating crucial factors involved in tissue regeneration, such as the expression of TGF-β1, VEGF, and runt-related transcription factor-2 (RUNX2) in periodontal ligament cells.^45^ Moreover, leptin is involved in bone metabolism as a pleiotropic adipokine.^49–51^ Leptin not only causes bone loss through hypothalamic relay, but also suppresses osteogenesis through the sympathetic nervous system via the central nervous system.^52^

Higher expression of visfatin was shown in patients with gingivitis and periodontitis compared with healthy people.^53^ In particular, visfatin secretion is stimulated by certain periodontitis-involved bacteria, such as *Porphyromonas gingivalis* and *Fusobacterium nucleatum*.^42^ Moreover, visfatin may accelerate periodontal inflammation and bone destruction via the production of MMP-1 and CCL2.^54^ Periodontal regenerative healing is also impaired by this adipokine, which downregulates the expression of growth factors crucial for periodontal repairs, such as VEGF, TGF-β1, collagen type 1 (COL1), POSTN and RUNX2.^55^

Elevated levels of resistin have been shown in patients with chronic periodontitis compared with clinically healthy controls.^56^ Moreover, research studies have indicated that resistin is involved in bone metabolism by decreasing the ratio of RANKL/OPG mRNA and stimulating IL-6 production.^57,58^

An association between reduced salivary omentin-1 levels and increased periodontal parameters was found in patients with chronic periodontitis.^59–61^ Additionally, higher salivary omentin-1 levels after non-surgical periodontal therapy were also concomitant with an improvement in the patient's periodontal health status.^59–61^ Finally, Figure 2 and *Table 1* show some of the main pro-and anti-inflammatory actions of the different adipokines contributing to periodontitis.

Due to a possible etiopathogenic relationship between periodontitis and metabolic-syndrome-related conditions, patients with severe periodontal disease should be screened for obesity, dyslipidaemia, glucose intolerance and hypertension. On the other hand, patients prone to or diagnosed with these diseases may also be referred to the dental clinic to check for and treat periodontal disease.^62^

## Conclusions

Adipokines are a large family of proteins secreted by the adipose tissue that perform different actions on inflammatory conditions and immunity. Certain inflammation-related diseases have been identified as risk factors for periodontitis; a clear example of this is obesity, where adipokine secretion is dysregulated in a complex process referred to as 'adiposopathy'. In patients with periodontitis, alterations in the levels of various adipokines, such as adiponectin, leptin, visfatin, resistin and omentin-1, which condition periodontal healing and bone loss, have been studied. Currently, patients with obesity, metabolic syndrome and diabetes should be referred for oral health evaluation to avoid associated complications. Finally, further studies are needed to unveil the potential prognostic and therapeutic target values of these molecules in periodontal diseases.
